# Exploiting the Interplay between Innate and Adaptive Immunity to Improve Immunotherapeutic Strategies for Epstein-Barr-Virus-Driven Disorders

**DOI:** 10.1155/2012/931952

**Published:** 2012-01-29

**Authors:** Debora Martorelli, Elena Muraro, Anna Merlo, Riccardo Turrini, Damiana Antonia Faè, Antonio Rosato, Riccardo Dolcetti

**Affiliations:** ^1^Cancer Bioimmunotherapy Unit CRO-IRCCS, National Cancer Institute, Via F. Gallini 2, 33081 Aviano, Italy; ^2^Department of Oncology and Surgical Sciences, University of Padova, Via F. Gallini 2, 35128 Padova, Italy; ^3^Cancer Bioimmunotherapy Unit, Department of Medical Oncology, CRO National Cancer Institute, Via F. Gallini 2, 33081 Aviano, Italy

## Abstract

The recent demonstration that immunotherapeutic approaches may be clinically effective for cancer patients has renewed the interest for this strategy of intervention. In particular, clinical trials using adoptive T-cell therapies disclosed encouraging results, particularly in the context of Epstein-Barr-virus- (EBV-) related tumors. Nevertheless, the rate of complete clinical responses is still limited, thus stimulating the development of more effective therapeutic protocols. Considering the relevance of innate immunity in controlling both infections and cancers, innovative immunotherapeutic approaches should take into account also this compartment to improve clinical efficacy. Evidence accumulated so far indicates that innate immunity effectors, particularly NK cells, can be exploited with therapeutic purposes and new targets have been recently identified. We herein review the complex interactions between EBV and innate immunity and summarize the therapeutic strategies involving both adaptive and innate immune system, in the light of a fruitful integration between these immunotherapeutic modalities for a better control of EBV-driven tumors.

## 1. Introduction

An increasing number of clinical trials involving cell-based immunotherapies is ongoing for the prevention and treatment of different types of human cancers [1–3]. These therapeutic approaches involve two different modalities: active cellular immunotherapies, that rely on autologous dendritic cells or other antigen presenting cells [4, 5] and adoptive T-cell therapies (ACT), in which the *ex vivo* activation, expansion, and subsequent reinfusion of tumor-reactive T cells hopefully result in attack and elimination of tumors [6–8]. Nevertheless, due to its complexity and costs ACT can be performed only by a limited number of specialized centers and it is still challenging to adapt this treatment for a broader use. Perhaps one of the best studied ACT approaches is constituted by the adoptive immunotherapy of EBV-associated malignancies.

**Figure 1:**
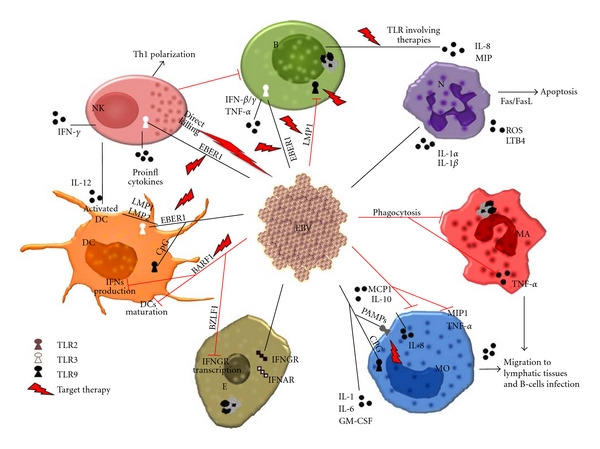
Interplay between EBV and innate immunity effectors. Main activating (dark lines) and inhibitory (red lines) effects of EBV infection on B lymphocytes (B), neutrophils (N), macrophages (MA), monocytes (MO), epithelial (E), dendritic (DC), and natural killer (NK) cells. Potential sites of intervention of targeted therapy approaches are also suggested (arrows). For further insights see text.

The Epstein-Barr Virus (EBV) is a double-stranded DNA virus, a member of the gamma herpes virus family and has a genome comprising approximately 172 kb pairs. Over 70 open reading frames allow for the transcription of genes for a large number of different viral proteins. Expression of different combinations of these proteins allows the virus to establish different forms of infection. Thus, in lytic infection, the virus expresses the full complement of immediate early, early, and late lytic cycle proteins and is capable of replicating within the host cell. In latent infection, the virus expresses a smaller number of proteins and does not replicate but is able to persist within the host cell. Four latency types have been described depending on which of these latent genes are expressed, as shown in Figure 1. Long-lived EBV-carrying memory B lymphocytes may express a putative Latency 0, characterized by complete silencing of the viral genome, or a Latency I, in which LMP-2A alone or together with EBNA-1 may be expressed [9]. The expression of these viral proteins is crucial for establishing and maintaining EBV persistence in memory B lymphocytes. An intermediate form of latency (Latency II) has been identified in B-lymphocytes homing to the germinal centers of lymphoid follicles [10]. In these cells, the expression of EBV proteins is restricted to EBNA-1 and the LMPs (LMP-1, -2A, and -2B), a “rescue” program that provides signals allowing infected lymphoblasts to survive and differentiate into memory B cells. In the absence of effective immune surveillance, as observed *in vitro *or *in vivo *in immune suppressed patients, EBV-infected B lymphocytes show a different latency program, called Latency III, characterized by the expression of the six EBV nuclear antigens (EBNA1-6) and the three LMPs [11]. During latency, EBV also expresses two small noncoding RNAs, known as EBER1 and EBER2 (167 and 172 nucleotides, resp.), the most abundant RNAs in EBV-infected cells [12]. Furthermore, a growing body of data indicates that viruses, including EBV, exploit the cellular microRNA (miRNA) machinery to modulate and/or subvert virus-host cell interactions [13–15]. miRNAs are 19- to 25-nucleotide-long single-stranded RNAs processed from transcripts with stem-loop structures [16] and endowed with diverse functions, including the regulation of cellular differentiation, proliferation, and apoptosis [17–19]. miRNAs might also function as tumor suppressors [20] or oncogenes [20, 21].

Primary EBV infection mainly takes place in the oropharyngeal region to which the virus is conveyed by saliva droplets from infected individuals [22]. The nature of the target cells in the oral mucosa is still controversial, but there is agreement that B cells are infected at some stage of the process. If infection is delayed to adolescence or adulthood, it can cause infectious mononucleosis (IM), a self-resolving lymphoid disorder largely resulting from an uncontrolled T-cell reaction directed against EBV-infected cells. In IM patients, EBV is exclusively found in B blasts that undergo continuous proliferation under the influence of all latent genes (Latency III). Following resolution of primary infection, EBV establishes a lifelong persistence in memory B cells where the virus usually remains clinically silent. In this B-cell reservoir, viral expression is entirely repressed, a process described as a “True Latency”. The pleiotropic nature of EBV target cells *in vivo*, including B and T lymphocytes, NK cells, squamous and glandular epithelia, and smooth muscle cells, demonstrates that the virus possesses the ability to infect a large spectrum of different cell types [23].

## 2. EBV-Associated Diseases

### 2.1. Infectious Mononucleosis

EBV is one of a variety of infectious agents that can induce an infectious mononucleosis syndrome. IM may be misdiagnosed as a high-grade large B-cell lymphoma, or even Hodgkin's lymphoma, particularly in cases containing a large number of B immunoblasts, some of which express CD30, and rare Reed-Sternberg like cells [24]. EBV-infected B-cells in IM show a typical Latency III expression pattern including EBERs, EBNA1, EBNA2, and LMP1, which renders virus detection very easy. However, the mere presence of EBV does not exclude a large B cell lymphoma, but detection of EBNA2 expression in infected cells strongly suggests the diagnosis of IM.

### 2.2. EBV-Associated Tumors in Immunosuppressed Individuals

The role of the immune system in controlling EBV latent infection is best illustrated by the observation that individuals with a congenital or acquired (e.g., after HIV infection or organ transplantation) immune deficiency are at increased risk to develop EBV-associated diseases, whose histological type varies according to the type and the severity of immunodeficiency. In these cases, the clinical context is more suggestive for the diagnosis of this category of tumors than the detection of EBV alone [25]. Accordingly, considering that the pattern of EBV latent gene expression may also vary concomitantly with cytomorphological changes [26], the assessment of viral status can have important consequences for the treatment of these lymphomas, as EBV-positive lesions are in principle amenable to EBV-specific T-cell therapy or even to antiviral therapy.

#### 2.2.1. Posttransplant Lymphoproliferative Disorders

Post-transplant lymphoproliferative disorder (PTLD) is thought to result from iatrogenic immune suppression and chronic antigenic stimulation from the engrafted organ after hematopoietic stem cell (HSC) or solid organ transplantation (SOT). PTLD includes a wide spectrum of diseases ranging from polyclonal reactive lymphoid hyperplasia to monoclonal malignant lymphoma. About 80% of PTLDs are associated with EBV and its overall incidence is about 1% in patients with hematopoietic cell transplantation and less than 2% in those with solid-organ transplantation. PTLD is more common in pediatric patients, largely because children are more likely to be primarily infected with EBV via the graft and the risk of developing PTLD is greatest during the first year after transplantation and declines thereafter [27]. After hematopoietic cell transplantation, this disease may involve nodal and extranodal sites, while PTLD after solid-organ transplantation frequently involves the allograft itself, which suggests a lymphomagenic role of chronic antigen stimulation occurring within the graft [28]. Other sites are also frequently involved, such as the gastrointestinal tract, liver, lungs, lymph nodes, bone marrow, skin, and central nervous system. Interestingly, PTLD occurring in the setting of primary EBV infection or associated with use of the immunosuppressive OKT3 antibody is often seen very early after the transplant in heart, kidney, and pancreas allograft recipients [29, 30]. Moreover, the reduction of pharmacologic load of immunosuppressive drugs may also lower the incidence of PTLD, by restoring EBV-specific immunity, provided that the risk of acute rejection of the allograft is closely monitored [31]. Finally, chemotherapy and anti-B-cell antibodies may be used in combination with the reduction in immune suppression, whereas surgery may be considered for localized PTLDs. Reduction of immune suppression alone results in clinical remission in 25–63% of adult and in 40–86% of pediatric PTLD patients [27].

#### 2.2.2. HIV-Associated Lymphomas

Patients with HIV infection are at 60 to 200-fold increased risk of developing various lymphomas, considered as the first acquired-immunodeficiency-syndrome-(AIDS-) defining illness in 3–5% of HIV+ patients [25]. EBV positivity is significantly correlated with HIV disease status and subtypes of HIV-associated lymphoma, generally involving extranodal tissues, in particular the gastrointestinal tract, lungs, liver, CNS, and bone marrow. The overall incidence of EBV-positive cells in HIV-related lymphomas is about 60%, even if the incidence varies with the site and subtype of lymphoma.

### 2.3. EBV and Hematological Malignancies

EBV infection has been associated with several hematopoietic cell tumors.

#### 2.3.1. B-Cell Lymphomas

Systematic testing of a large panel of B-cell lymphomas has shown that the EBV genome is found mainly in high-grade lymphomas, though rare cases of partial EBV infection of low-grade B-cell lymphomas have been observed [32].

**Burkitt Lymphoma (BL)**

Burkitt lymphoma was first described in children from equatorial Africa by Denis Burkitt in 1958. It is a highly proliferative B-cell tumor that includes 3 variants: *endemic *(affecting children in equatorial Africa and New Guinea), *sporadic *(children and young adults throughout the world), and *immunodeficiency-related *(primarily in association with HIV infection) [33]. EBV has been detected in virtually all cases of the endemic variant, in 15–20% of the sporadic variant, and in 30–40% of the immunodeficiency-related variant [34]. In all variants, irrespective of the EBV status, constitutive activation of the c-myc oncogene through its translocation into one of the immunoglobulin loci is the key factor in the oncogenesis of BL [33, 34]. The detection of somatic hypermutations in the V region of the immunoglobulin genes and the phenotype of Burkitt lymphoma cells indicate a germinal center cell origin of this lymphoma [35]. Most EBV-positive cases exhibit a highly restricted pattern of expression of latent gene products, including only EBNA1 and the EBERs (Latency I). However, it has been recently reported that some cases, in addition to EBNA1 and the EBERs, also express EBNAs 3A, 3B, and 3C, but still lack EBNA2 and the latent membrane proteins [36]. This peculiar restricted latency pattern has stimulated an intense debate about the role of EBV in the pathogenesis of Burkitt lymphoma. A number of reports demonstrate that EBNA1 plays a crucial role in the maintenance and replication of the viral genome, but its oncogenic potential is still highly controversial [34, 37]. As the EBERs are believed to possess antiapoptotic activities, as well as the ability to induce the expression of IL-10, which may promote tumor cell growth and survival, it has been suggested that these RNAs may play an essential role in the oncogenesis of Burkitt lymphoma [38]. Moreover, analysis of the chromosome breakpoints in the myc-activating translocations indicates that these genetic changes have occurred during either somatic mutation or class switch recombination, which are both unique processes of germinal centre cells [39]. In this regard, recent findings demonstrated that peculiar subsets of endemic BLs show the presence of an EBNA2-deleted virus genome, resulting in a high Wp promoter activity and a constitutive BHRF1 expression. These findings confirm previous data obtained in mouse models of c-myc-driven lymphomagenesis [40], where full malignant transformation occurs only when a target cell expressing a deregulated c-myc oncogene acquires complementary changes that counteract c-myc-driven apoptosis [41]. It has been suggested that BHRF1 may be such complementary factor, thus providing the first evidence implicating a herpesvirus bcl2-like protein in viral oncogenesis [42].

**Hodgkin's Lymphoma (HL)**

Hodgkin's lymphoma is the most common EBV-associated lymphoma in Europe and USA, with about 40% of EBV-positive HL cases [35]. Interestingly, in the tumor tissue, only a small subset of cells, the so-called Hodgkin-Reed-Sternberg (HRS) cells, are the EBV-transformed tumor cells, primarily of B-cell origin [43], while the majority of cells composing the tumor mass are infiltrating lymphocytes (TILs). This indicates that HL has already managed to generate an immunosuppressive environment that allows tumor cells to grow despite extensive homing of immune cells to the tumor site. Moreover, HRS cells have been shown to produce immunosuppressive cytokines, including IL-10, IL-13, and TGF-*β* [44, 45] and to allow tumor immune escape by several mechanisms. As a result, regulatory T (Treg) cell populations are enriched in HL tissues and are able to strongly suppress peripheral blood cell proliferation and cytokine secretion [46]. In addition to this local immune suppression, selective systemic impairment of EBV-specific T-cell responses might also contribute to HL development. Along these lines, HL patients have diminished EBNA1 specific CD4^+^ T-cell responses, while they maintain CD8+ T-cell responses against other latent and lytic EBV antigens [42]. These findings suggest that immunotherapeutic approaches should be developed to correct both the selective systemic immune impairment and tumor microenvironment specific deficiencies in EBV-specific immune control. Since the tumor cells seem to have no defects in antigen processing for MHC class I (MHC-I) presentation, interventions able to correct the selective systemic loss of EBV specific T-cell responses and to overcome the local immune suppression in the tumor tissue should be explored as treatments of this EBV-associated malignancy and ideally such modalities could be used for prevention in high-risk populations.

#### 2.3.2. Tumors Derived from T and NK Cells

The rate of association of the virus with these tumors is so high that the absence of the virus virtually excludes diagnosis. All described entities, mostly encountered in Japan and South-East Asia, are closely related and can also arise in a single patient.

**Peripheral T-Cell Lymphoma, NK Tumors, and EBV-Associated Haematophagocytic Syndrome (HS)**

HS is frequently observed in infants with immune deficiencies in association with primary or chronic EBV infection. In these patients, T-cell lymphocytosis and EBV-positive T cells are frequently observed in the blood and tissues. Affected individuals are at high risk of subsequent or concurrent development of EBV-positive T-cell lymphomas and NK cell tumors [23]. Detection of EBV in either normal T lymphocytes or tumor cells establishes the diagnosis.

**NK Leukaemias**

NK leukaemias are mainly observed in adolescents of the Far East in association with hepatosplenomegaly and multiple cutaneous lesions [24]. Histological examination identifies large granular lymphocytes expressing NK markers. The rate of EBV association approaches 100%.

**Extranodal NK/T Cell Lymphoma (NKTCL)**

Extranodal NK/T cell lymphoma of the nasal type consists of an angioinvasive and angiodestructive lymphoid infiltrate with necrosis that affects and can cause destruction of the nasal cavity and of several anatomical structures of the midface [47]. Extranodal NKTCL represents the major group of mature NK cell neoplasms in the recently revised WHO classification of hematolymphoid tumors [48]. Importantly, EBV is present in virtually all NKTCL cases (EBER-positive, EBV proteins rarely expressed, with a more or less complete latency II pattern) [49]. Circulating EBV DNA load is an important prognostic factor in this setting, and plasma EBV DNA levels can also be used for disease monitoring [50]. These tumors are more frequently observed in Asian and Central and South American populations, where they can account for up to 10% of all non-Hodgkin's lymphomas [51].

**Inflammatory Pseudotumor-Like Follicular Dendritic Cell Tumor (IPLFD)**

IPLFD preferentially develops in liver and spleen, or more rarely in lymph nodes. This exceptional tumor is characterized by a prominent inflammatory background including numerous lymphocytes and plasma cells, among which neoplastic follicular dendritic cells (FDCs) can be identified [52]. Viral infection is readily established with an EBER assay showing intense signals in all cancer cells; LMP1 staining, although consistently positive, is generally weak. Due to the consistent association with EBV, diagnosis of this entity requires detection of the virus.

### 2.4. EBV and Nasopharyngeal Carcinoma (NPC)

Nasopharyngeal carcinoma is classified as a malignant neoplasm arising from the mucosal epithelium of the nasopharynx, most often within the lateral nasopharyngeal recess or fossa of Rosenmüller. There are three histopathologic subtypes of NPC: a well-differentiated keratinizing type, a moderately-differentiated, nonkeratinizing type, and an undifferentiated type, which typically contains large numbers of noncancerous chronic inflammatory lymphocytes. The undifferentiated form is the most common and also the most strongly associated with EBV infection. NPCs are rare in most countries, especially in Europe and North America (incidence below 1/100,000), whereas it has a high incidence in several areas in Southern China, especially in the Cantonese region around Guangzhou, where the incidence is approximately of 30–80/100,000 cases per year and where the diet probably plays a carcinogenic role. EBV is strongly associated with undifferentiated nasopharyngeal carcinomas, in which virus infection is observed virtually in 100% of the cases [53], and epithelial EBV infection is also well documented *in vitro* [54]. Accordingly, following primary infection in the oropharynx, EBV persists in numerous anatomical sites including pharyngeal tonsils, adenoids, lymph nodes, and peripheral blood, where the virus is present in small resting memory B cells with latent gene expression being virtually absent or limited to EBER, LMP2a, and EBNA1 [55]. Despite available evidence, the presence of EBV within normal epithelial cells *in vivo* remains a matter of debate. Some authors demonstrated the presence of EBV in desquamated squamous and tonsillar epithelium [56, 57]. In contrast, other studies have reported no virus in the same cells or unequivocal evidence of EBV-infected tonsillar epithelial cells by EBER ISH and/or LMP1 immunostaining [58–60]. Although the demonstration of virus latency and replication *in vivo* is still under investigation, it is well known that vigorous humoral and cellular immune responses control the proliferation of EBV-infected cells in healthy virus carriers [61, 62]. Early after the discovery of EBV association with NPC, a deregulation of the EBV-specific immune response with elevated IgA titers against the virus was noted [63]. This indicated that the immune response at the site of tumor development was abnormal, and that the tumor might influence its microenvironment to facilitate neoplastic cell growth. Accordingly, recent studies supported the notion that local immune suppression rather than a systemic deficiency in EBV-specific immune control may contribute to NPC development. Indeed, both nonspecific (NK-cell-mediated) and EBV-specific (T-cell-mediated) responses were shown to play important roles during primary infection, while EBV-specific T-cells appear to be critically involved in restraining the proliferation of EBV infected cells during life-long persistent infection. On these grounds, some studies confirmed that EBV-specific T cells are maintained in healthy carriers at relatively high frequencies throughout life [64], whereas EBV-specific CD4^+^ and CD8^+^ responses might be reactivated from peripheral blood of NPC patients [65, 66]. In particular, clinical and immunologic responses were observed by Comoli et al. after EBV-targeting CTL therapy in patients with radiotherapy- and chemotherapy-resistant stage IV NPCs [67]. Importantly, they confirmed that, despite immunosuppressive treatments, it is possible to reactivate and expand *ex vivo *these effectors, suggesting that reconstitution of EBV-specific immunity could also be a useful strategy in the management of NPC. Interestingly, a significant immune suppression can also be observed at the tumor site. Indeed, even though LMP1- and LMP2-specific CTLs were enriched in tumor infiltrating lymphocytes, their cytotoxicity and cytokine secretion could be impaired, resulting in a local immunomodulation. Particularly, the high presence of natural Tregs (CD4^+^CD25^+^FoxP3^+^) [68] and the production of galectin-9-carrying exosomes in NPC patients [69] could suppress EBV-specific immune responses in tumor tissues. In addition to active T cell suppression at the tumor site, the expression of immune suppressive cytokines, such as interleukin 10 (IL-10), has also been related with an immune escape mechanism, partly because EBV-encoded LMP1 can induce IL-10 [70]. However, the importance of immune suppressive cytokines seems limited in NPC [71]. In addition, the efficiency with which NPC can present antigens to T-cells might also be compromised. While earlier studies based on a limited number of NPC cell lines suggested that antigen processing for MHC-I presentation was intact in NPC cells [72], a more recent study on primary tumor tissues suggested that the MHC-I antigen processing machinery is downregulated in the majority of tumors [73]. Together, these data suggest that NPC cells impair EBV-specific immune control locally, while allowing efficient systemic immune responses against this virus.

## 3. EBV Infection and the Immune System

The immune system preserves the integrity of its host by recognizing and resisting invaders; the frequency and severity of EBV-associated diseases, particularly in immunocompromised individuals, highlight the critical importance of immune responses in controlling this virus infection. It is well known that the adaptive arm of the immune system plays a pivotal role in protective immunity against EBV [74, 75]. Nevertheless, emerging data suggest that also the innate compartment may cooperate to the immune response [76–78], mounting a highly coordinated cascade of molecular interactions, which not only activate but also heighten the potential to bias the ensuing adaptive phenotype. Since the role of the innate immunity in EBV infection is still unclear, our aim is to mainly review current knowledge in this respect, with a particular focus on the contribution of innate immune cells in controlling the progression of virus infection towards the oncogenic phenotype.

### 3.1. Innate Immunity and EBV Infection

Understanding the importance of host innate immune responses in the context of EBV infection will allow a better understanding of how EBV can persist in the infected host to promote cell transformation [79]. Intriguingly, EBV interacts with different cell types that are crucial mediators of innate immune responses (Figure 1), including NK cells, neutrophils, and monocytes/macrophages. Moreover, the virus can also infect epithelial cells that play an important role in the innate resistance to different pathogens [78].

#### 3.1.1. NK Cells

NK cells are innate lymphocytes that play a pivotal role in the control of infections and in the immune surveillance against transformed cells [80]. In particular, early after primary viral infections NK cells are thought to limit the viral burden until virus-specific T cells are able to eliminate the infection or control viral titres at low levels. Notably, NK cells produce cytokines such as interferon-*γ* (IFN-*γ*), proliferate, and increase their cytotoxicity upon activation by both myeloid and plasmacytoid dendritic cells (DCs) [81]. Therefore, DCs seem to activate NK cells early after infection in order to restrict pathogen replication until the adaptive immune system establishes long-lasting immune control. Two main functional NK cell subsets have been distinguished in humans: CD16^+^CD56^dim^ NK cells, which readily lyse susceptible target cells, but secrete only low levels of cytokines after activation; and CD16^−^CD56^bright^ NK cells that produce large amounts of cytokines upon stimulation and acquire cytotoxicity only after prolonged activation [82]. CD16^+^CD56^dim^ cells constitute about 90% of human circulating NK cells, whereas CD16^−^CD56^bright^ NK cells represent <10% of the peripheral blood NK cell pool. However, in secondary lymphoid organs such as tonsils and lymph nodes, the CD16^−^CD56^bright^ NK cells are the dominant subset [83]. Interestingly, viral double-stranded RNA (dsRNA) products, including EBV convergent transcription of LMP1 and LMP2 RNA and EBERs dsRNA stem-loop structures [84], trigger monocyte toll-like receptors (TLRs) inducing maturation of DCs, which through the production of high levels of IL-12, activate CD16^−^CD56^bright^ NK cells more efficiently than other mature DC preparations [85]. Because the tonsils are the primary site of EBV infection, the DC-NK cell crosstalk could trigger NK cells to limit B cell transformation during EBV infection (Figure 1). Indeed, *in vitro* virus-activated DCs elicited 50-fold stronger IFN-*γ* secretion from tonsil NK cells than from peripheral blood NK cells; these high IFN-*γ* concentrations delayed latent EBV antigen expression, inhibit B-cells transformation, and decrease their proliferation during the first week after EBV infection *in vitro *[86]. In addition to its direct antiviral activity, IFN-*γ* secreted by DC-activated NK cells might also shape the EBV-specific adaptive immune response by favouring a Th1-polarization (Figure 1), which is observed in EBV-positive individuals. These *in vitro* findings suggest that humans have a strategically well-positioned population of NK cells, which directly hampers pathogen entry at mucosal sites and might restrict EBV infection until adaptive immunity establishes immune control of this persistent and oncogenic human pathogen [86]. However, only few *in vivo *examples support this hypothesis [85], and little is known about NK cell contribution to EBV persistence, thus still leaving several open questions.

In addition to protective T-cell immunity in healthy virus carriers, several lines of evidence suggest a role for innate lymphocytes in the resistance against EBV-associated malignancies. For example, in male patients with X-linked lymphoproliferative disease (XLP), who frequently succumb after primary EBV infection as a consequence of EBV-driven lymphomas, a mutation in the SAP (signalling-lymphocyte activation-molecule-(SLAM-) associated protein) gene leads to defective recognition of EBV-transformed B cells by T and NK cells [87]. Although the biologic effects of SAP mutations are not limited to alterations in NK cell function, this defective recognition most likely contributes to loss of EBV specific immune control. Furthermore, during EBV latency, the virus develops several mechanisms of immunoescape from this innate immunity cell subset, including the inhibition of NK cells activation through Epstein-Barr-virus-induced gene 3 (EBI3). EBV-transformed B-lymphocytes express at high levels the EBI3 protein, which is related to the p40 subunit of IL-12 and associates with p35 and, consistent with a potential immunosuppressive activity, a peptide derived from EBI3 has been shown to bind HLA-G, which is the ligand of inhibitory receptors expressed on NK cells and CTLs [78, 88]. Moreover, *in situ *analysis revealed EBI3 overexpression in neoplastic cells of individuals affected by HLs and LMP1-positive EBV-associated lymphoproliferative disorders (including posttransplant LPDs and nasal-type NK/T-cell lymphomas) [89], thus supporting its hypothetical role in regulating immune response against EBV. Finally, NK cells seem to play a fundamental role in T-cell-depleted HLA-haploidentical HSCT (hematopoietic stem cell transplantation), indeed in the early posttransplant period, lymphocytes in the peripheral blood are mostly NK cell populations and the antileukemia effect is mediated by “alloreactive” (i.e., KIR/HLA-mismatched) NK cells originated from donor HSCs [90]. Nevertheless, in these patients an increase in EBV load is often observed, until they receive donor EBV-specific cytotoxic T-lymphocytes, suggesting that in this setting NK cells are not able to control EBV DNAemia [91].

#### 3.1.2. Phagocytes

In addition to the clearance of virions or virally infected cells by phagocytosis, both neutrophils and monocytes elicit a respiratory burst when exposed to viruses and can produce a variety of mediators, some of which have antiviral activity [92]. Moreover, EBV genome was detected in non-B-cells, including phagocytes, of most patients with EBV-lymphoproliferative disorders [93]. Interestingly, monocytes and macrophages are involved in the uptake of small vesicles called exosomes, containing viral miRNA. Exosomes are nanosized membrane vesicles released from a wide variety of cells that have immune stimulatory, inhibitory, or tolerance-inducing effects, depending on their cellular origin [94]. Exosome secretion protects miRNA by RNases degradation, thus miRNA secreted by EBV-infected cells are transferred to uninfected cells and could act also in EBV^−^ recipients [95]. It has been demonstrated that exosomes of different origin and with function can selectively target different immune cells *in vitro*. In this regard, while exosomes derived from EBV-transformed lymphoblastoid B-cell lines target preferentially B cells, DC-derived exosomes are mainly associated with monocytes, which actively engulf these vesicles [94]. Thus, exosomes play a role during the early phases of EBV infection, also involving innate immunity-related cell types, that otherwise would not be directly targeted by the virus. We will now consider separately the main subsets of “professional” phagocytes and their interplay with EBV.

**Neutrophils**

As in the case of most viral infections, a blood neutrophil increase is observed during the initial phases of EBV infection, whereas a transient episode of acute neutropenia is often seen in IM patients during the third and fourth weeks of illness [96–98]. *In vitro* studies showed that the interaction of EBV with these cells causes an abortive infection, where the virus penetrates the cells and is transported to the nucleus, even if this is not followed by gene expression. Infected neutrophils rapidly die by apoptosis, probably via Fas triggering by its ligand that is upregulated upon EBV infection [99]. EBV particles can bind to 30% of neutrophils, although these cells do not express the canonical EBV receptor CD21 [100], and this binding is sufficient to trigger the release of inflammatory mediators. Moreover, EBV induces neutrophils to produce IL-1*α* and IL-1*β* (Figure 1), but this is counteracted by a considerably stronger induction of IL-1R antagonist that neutralizes the proinflammatory activity of IL-1 [77]. IL-1Ra to IL-1 ratio of 10–100 is necessary to inhibit the IL-1 effects on target cells by 50% [101]. In response to EBV infection, the levels of secreted IL-1Ra in neutrophil culture supernatants are approximately 3600 and 610 times more than those of IL-1*α* and IL-1*β*, respectively [102], an indication of a predominant IL-1Ra response. By favouring the secretion of IL-1Ra, EBV may thereby shift the tightly regulated balance between IL-1 and IL-1Ra production. Expression of the chemotactic factors IL-8 and macrophage inflammatory protein (MIP)-1 was also detected in EBV-infected neutrophils [103]. The production of these cytokines by neutrophils could be involved in the recruitment of leukocytes in the lymphoid tissues and allow EBV a direct access to B cells. Two additional biological functions of neutrophils are known to be primed by EBV: the biosynthesis of leukotriene (LT) B4 and the production of reactive oxygen species [77]. LTB4 exerts a wide array of immunoregulatory activities (Figure 1). Among others, it stimulates locomotion and chemotaxis of phagocytes, modulates lymphocyte as well as phagocyte functions and is also involved in the regulation of cytokines production [104]. In addition, LTB4 enhances antitumor activities of NK cells and was recently reported to also exert antiviral activities [105]. Production and release of reactive superoxide anion, also known as respiratory burst activity, is another important function involved in destruction of viral particles. The fact that neutrophils become more responsive to the release of LTB4 and O_2_^−^ anion after interacting with EBV constitutes an alternative route for the immune defence to counteract viral invasion [77]. Therefore, the interaction of EBV with neutrophils has opposing influence on the initiation of immunity. Some of these effects such as the secretion of IL-1, IL-8, MIP-1*α*, LTB4, and reactive superoxide anion, promote the development of EBV-specific immunity, while the upregulation of IL-1R and the induction of apoptosis in neutrophils inhibits anti-EBV immune responses [78].

**Monocytes, Macrophages, and Dendritic Cells**

In antiviral immune response, similarly to neutrophils, monocytes are rapidly recruited to sites of viral infection and were shown to adhere to flu-infected cells [106]. Monocytes are also known to play a key role in the initiation of specific immune responses. Moreover, they may contribute to host defence by mediating antibody-dependent cellular cytotoxicity (ADCC) via activation of NK cells. Episodes of monocytopenia were observed during the acute phase of IM [107]. In addition, patients with EBV-associated HL often show a deficiency in monocyte-mediated ADCC, suggesting that monocyte functions are affected during the course of EBV infection [77], as also demonstrated by the reduced phagocytic activity observed in EBV-infected monocytes [108]. This effect was probably caused by the impairment of protein kinase C (PKC) activity. EBV infection was found to prevent PMA-induced translocation of PKC and by altering the subcellular localization of the receptor for activated C kinase (RACK) 1 [109]. RACK1 is an essential anchoring protein that interacts and translocates with activated PKCs in response to a stimulus. Interactions of RACK1 with the EBV-encoded ZEBRA protein may inhibit PKC translocation and activation [77]. EBV infection inhibits also the functional ability of macrophages to respond to bacterial challenge [110] by reducing their phagocytic potential. The alteration of the phagocytic process is just an example of the EBV versatility exhibited to target the multiple facets of monocytes and macrophages antiviral activities.

Moreover, EBV directly binds the surface of approximately the 20% of monocytes, involving cellular receptors that are still not well characterized. Entry and translocation of EBV particles into the nucleus were visualized by electron microscopy and confirmed by the detection of EBV genome in isolated nuclei [108]. Interestingly, nuclear levels of viral genomic DNA were found to increase with time, suggesting that replication may actually occur in monocytes. Furthermore, although EBV propagation is mainly due to cell-cell contact that primarily involves B cells and epithelial cells, also monocytes and macrophages may participate in the transport of the virus and favour its propagation [111], especially within the oral epithelium, where EBV actively replicates and is released into the saliva [112]. The outcome of monocyte/EBV interaction is strongly influenced by the stage of differentiation of monocytes. Indeed, EBV infection inhibits the development of DCs from monocyte precursors [113] and the exposure to EBV induces apoptosis of a large proportion of monocytes cultured in the presence of GM-CSF and IL-4. Binding of EBV to monocytes through the viral membrane fusion complex may be sufficient to trigger events that will eventually lead to cell death. By inhibiting the differentiation of monocytes into mature DCs, EBV temporarily halts the onset of immune responses during primary infection, creating a time window for efficient viral replication. This permits the accumulation of a large pool of virus infected B-lymphocytes and allows their access to the memory B-cell compartment, interferes with the functions of DCs during the initiation of virus-specific immune response, and modifies the profile of secreted cytokines in order to create a favourable environment for viral propagation [77].

In this context, EBV has evolved over the millennia to modulate the cytokine network of the host in order to be not completely eliminated after primary infection and to promote its life-long persistence. Purified gp350/220, the major glycoprotein component of EBV envelope, induces secretion of IL-1, IL-6, and TNF-*α* by monocytes [77]. In this regard, during the course of primary infection, IL-1 and IL-6 are frequently detected in the serum and in tonsils of IM patients [114]. In addition, both IL-1 and IL-6 are actively secreted by EBV-immortalized B cells and have been shown to act as autocrine/paracrine growth factors for these cells (Figure 1). TNF-*α* can increase macrophage cytotoxicity, mediate natural cell cytotoxicity, and also participate in the regulation, maturation, activation, and lymphocytes proliferation. TNF-*α* was also reported to elicit antiviral activities against both RNA and DNA viruses (reviewed in [77]). Moreover, recombinant TNF-*α* was found to inhibit, in a dose-dependent manner, the proliferation and differentiation of both EBV-activated and- transformed human B cells [115]. Therefore, it is not at all surprising that levels of TNF-*α* are detected in the serum of IM patients [114], an indication of the importance of this cytokine in the ongoing antiviral response. However, the entire virus inhibits the secretion of TNF-*α* by monocytes and macrophages, and, interestingly, neither peripheral blood mononuclear cells nor purified monocytes showed increased secretions of TNF-*α* in response to EBV [77]. EBV downregulated the cellular levels of TNF-*α* mRNA transcripts, suggesting that it may elicit its suppressive action at the transcriptional level. Furthermore, the virus inhibited the synthesis of the chemokine MIP-1*α*, thus reducing the recruitment of leukocytes. EBV was also found to induce the production of granulocyte-macrophage colony-stimulating factor (GM-CSF) upon adsorption to the cell surface of monocytes [116]. Secretion of GM-CSF by monocytes was shown to synergize with EBV to enhance release of IL-8, MIP-1, and IL-1 by neutrophils (Figure 1), as well as the magnitude of EBV priming effect on superoxide and LTB4 production. EBV differentially affected the production of leukotrienes and prostaglandins (PGs) in monocytes. The direct interaction of EBV gp350 with the cell surface is sufficient to prime monocytes for an enhanced release of LTB4. A completely opposite effect is extended on PGs production [117]. PGs, such as PGE2, are potent vasodilators, hyperalgesic agents, and also pyrogenic substances. Both IL-2 and IFN-*γ* production by Th1 cells were downregulated by PGE2 via increased levels of cAMP [118]. In contrast, PGE2 favours Th0-like response to shift towards a Th2-like pattern and promotes the synthesis of IL-4, IL-5, and IL-10 by these cells [119]. In addition, PGE2 can regulate humoral responses by increasing the immunoglobulin class switching, particularly IgE in B lymphocytes. PGE2 is also known to exert antiviral activities both *in vitro* and *in vivo*. EBV suppresses the production of PGE2 by downregulating the LPS-induced expression of COX-2, without altering that of constitutive COX-1. Finally, the lytic gene BARF1 neutralizes the proliferative effects of CSF-1 on monocytes and inhibits poly(I-C)-induced secretion of INF-*α* by these cells [120], interfering with IFN-dependent antiviral effects of innate immunity [78]. These observations show that EBV has developed a perfect way of rapidly promoting the expression of its own genes while simultaneously shutting down the transcriptional programs of the cells.

#### 3.1.3. Epithelial Cells

Epithelial cells have an important role in the innate resistance to different pathogens and represent a preferential target of EBV infection, even if i*n vivo *analysis revealed no evidence of EBV infection in salivary glands and only rare positivity in normal squamous epithelial cells at the tongue margin [121]. In this regard, kinetics studies performed in healthy individuals underlined the potential importance of epithelial cells in EBV replication and shedding, assuming that B cells sporadically release virus that infects epithelial cells, which in turn may contribute to amplify the amount of shed virus; thus the mouth is probably not a reservoir of the virus, but a conduit through which a continuous flow stream of virus passes in saliva [122]. However, since EBV can be detected in most undifferentiated NPC and in precancerous lesions, EBV infection has long been postulated to play an important role in NPC pathogenesis. It is worth mentioning in this respect that an *in vitro *model of EBV infection in premalignant nasopharyngeal epithelial cells was described by Tsang and colleagues [123]. The direct infection of epithelial cells requires the interaction between EBV glycoproteins gHgL and cellular *ανβ*6 and *ανβ*8 integrins [124]. In addition, an alternative process named “transfer infection” was proposed to explain the interplay between EBV and epithelial cells: the infection of memory B cells determines CD21-mediated capping of virus and activation of adhesion molecules, which in turn facilitates conjugate formation between B cells and epithelial cells and then the subsequent entry of EBV into epithelial cells [125]. EBV-positive cells, such as EBV-infected B-cells and EBV-carrying carcinoma cells, secrete exosomes enriched in LMP1 [126], which may interact with surrounding cells, in particular suppressing neighbouring T cells [127], thus contributing to immune modulation [128]. Moreover, in EBV-infected epithelial cells LMP2A and LMP2B accelerated the turnover of IFN receptors through processes requiring endosome acidification [129]. This function forms part of EBV strategy to limit antiviral responses. Interferons constitute a first line of defence against viral infection, serving to integrate innate and adaptive immune responses and limiting the replication and spread of viruses. IFN*α* and IFN*β* (type I IFNs) block viral replication by inhibiting cellular protein synthesis, stimulating the expression of proinflammatory cytokines and orchestrating cellular immune responses. Similarly, IFN-*γ* (type II IFN) has a key function in “fine-tuning” the adaptive immune response. Whereas IFN-*α* and IFN-*β* share a common receptor, IFNAR, IFN-*γ* binds to IFNGR. The BZLF1 protein of EBV has been reported to downregulate transcription of the IFNGR during viral entry into lytic cycle, thereby protecting lytically infected cells from immune surveillance. Similarly, LMP2A and LMP2B attenuate IFN responses by influencing IFNAR and IFNGR stability (Figure 1), promoting their ubiquitination and the trafficking from endosomes to lysosomes. The ability of these viral proteins to modulate IFN signalling is another example of a successful viral strategy for circumventing the innate immune response, an effect that is generally exploited in persistent virus infections but, in the context of oncogenic viruses such as EBV, may also contribute to tumor development [129].

#### 3.1.4. Toll-Like Receptors

Innate immunity uses several pattern recognition receptors to sense pathogen-associated molecular patterns (PAMPs). The TLRs are the most studied pattern recognition receptors, and activation by pathogen components leads to robust immune response. TLR activation has probably multiple downstream effects during primary EBV infection, some of which may favour viral latency or reactivation and other may facilitate immune control. TLR2 was reported to recognize several PAMPs conserved on virions from different members of human herpesviruses. TLR9 is an important receptor for nucleic-acid-containing unmethylated CpG motifs present in both bacterial and viral DNA, whereas TLR7 senses single-stranded RNA [130]. A rapid detection of EBV particles during primary infection and the detection of secreted components or reactivated virus from EBV-infected cells are crucial for an efficient control of EBV infection. Intact viral particles are recognized by the membrane surface TLR2 (Figure 1) [131], likely through the interaction with gp350, the major envelope glycoprotein which mediates also EBV entry into B cells [132]. In addition, TLR2 is involved in the recognition of the unstructural protein dUTPase, which determines the activation of NF-*κ* B via MyD88-dependent signalling cascade and induces expression of proinflammatory cytokines in macrophages [133]. Following viral entry into the cells, viral DNA is subsequently recognized by TLR9 (Figure 1). Such dual interactions, through TLR2 on cell membrane and by intracellular TLR9, lead to a rapid production of IL-8 to initiate an effective immune response. Production of MCP-1 and IL-10 by monocytes in response to EBV is mainly dependent on TLR2 rather than TLR9 [134]. Therefore, because monocytes express low levels of TLR9, it seems plausible that they have acquired the ability to use one TLR, in this case TLR2, to produce different cytokines to better respond to EBV before its entry into the cell. On the contrary, plasmacytoid DCs (pDCs) seem to recognize the virus primarily through TLR9 [134], which, in combination with TLR7 activation, could potentiate the production of type I IFNs via IRF7, promoting the activation of NK cells and IFN-*γ* producing CD3^+^ T cells [135]. Such recognition by a single cell type through multiple TLRs could then ensure an efficient innate response, but may also optimize the detection of the virus once it has escaped recognition by membrane TLR or intracellular TLR following entry into the cytosol. However, EBV has developed the ability to escape the innate immune response by downregulating TLR9 transcription [110]. Notably, the virus exerts this activity by using its major oncoprotein LMP1 via activation of NF-*κ*B signalling. Primary B-cell infection by EBV resulted in an impairment of TLR9 pathway functionality, correlated with a decrease of TLR9 mRNA and protein levels in comparison with noninfected primary B cells [79]. In addition to TLR9, Fathallah et al. observed that EBV infection of primary B cells led to inhibition of TLR2 transcription and functionality [79]. Previous data showed that the major oncoproteins, E6 and E7, from the high-risk HPV type 16 inhibit the expression of TLR9. Thus, the ability to downregulate TLR9 expression is probably a shared property among DNA tumor viruses [136]. Interestingly, the low-risk HPV types, associated with benign cervical lesions, are unable to alter TLR9 expression, suggesting that TLR9 downregulation is an event exclusively associated with carcinogenic viruses [79].

The EBV noncoding RNAs, EBV-encoded RNA 1 (EBER1), and EBER2 are expected to form dsRNA-like structures [137]. EBER is the most abundant viral transcript in latently EBV-infected cells and binds to several cellular proteins including RNA-activated protein kinase (PKR), ribosomal protein 22 (L22), lupus erythematosis-associated antigen (La), and retinoic acid inducible gene I (RIG-I; reviewed in [138]). EBER exists in the sera of patients with active EBV infections and induces type I IFNs and inflammatory cytokines through TLR3-mediated signalling, which is a sensor of viral double-stranded RNA (dsRNA) [84]. This may account for the pathogenesis of active EBV infections that are characterized by cytokinemia. Furthermore, EBER1-treated DCs may induce primary immune responses, suggesting that during active infection, EBER1-mediated TLR3 stimulation is responsible for immune activation by EBV. TLR3 is predominantly expressed by DCs, but also by CD8^+^ T cells and NK cells, thus, circulating EBER1 could induce the activation of both DCs and T cells (Figure 1) [84]. These findings suggest that immunopathologic alterations that are caused by active EBV infections could be attributed to TLR3-mediated cytokinemia induced by EBER1, and that circulating EBER1 could be a potential target for therapeutic agents [84]. Moreover, EBERs may have several roles in EBV-driven tumorigenesis: EBER1 and EBER2 contribute to the clonal proliferation of EBV-negative BL cells in soft agar, promote tumorigenicity in SCID mice, upregulate the bcl-2 oncoprotein, induce resistance to apoptosis, and favour the maintenance of malignant phenotypes in BL cells. EBERs induce the expression of IL-10 in BL cells, insulin-like growth factor 1 (IGF-I) in gastric and NPC cells. In BL cells, EBERs prevent IFN-*α*-mediated apoptosis, while in epithelial cells these RNAs confer resistance to Fas-mediated apoptosis by blocking PKR activity. EBERs also play critical roles in the growth transformation of B lymphocytes [84, 138].

EBV is also able to induce the expression of cellular miRNA [131], in particular LMP1 signalling, through NF-*κ* B and AP1, stimulates the expression of cellular miR-155, which in turn targets intermediates of innate immune signalling pathways [139]. In addition, during latency, the virus induces miR-146, involved in the attenuation of type I IFN production in macrophages [140], and miR-21 that negatively regulates TLR4 signaling [141].

Given the fact that B-cell activation and proliferation require the stimulation of TLR, it is possible that TLR ligand-bearing pathogens, such as bacteria colocalized at sites of EBV infection or replication (i.e., in tonsillitis during infectious mononucleosis), might favour EBV establishment and spread by increased proliferation of infected B cells. Similarly, bacterial or fungal coinfection could influence and possibly aggravate PTLD by triggering TLRs on EBV infected B cells and thereby supporting their proliferation or modifying lytic EBV reactivation. Likewise, microbial infection may be a cofactor for EBV-associated BL, which mainly occurs in regions where malaria is endemic. EBV itself is a source of endogenous ligands of TLRs and other pattern recognition receptors (PRRs) and might be capable of triggering TLR9, mediated by hypomethylated CpG motifs in the viral DNA genome [142].

Understanding TLR implications during early and acute phases of EBV infection must therefore be investigated to further develop strategies to treat EBV infection, especially in immunosuppressed or autoimmune disease patients, in which EBV was suggested to exacerbate clinical symptoms [134].

### 3.2. Adaptive Immunity and EBV Infection

The recognition of antigens and their proper presentation and processing lead to the activation of specific adaptive immune responses that ultimately control the invading infectious agents. The central feature of immune memory is the ability of memory cells to mediate faster, stronger, and more effective responses to secondary pathogen challenges than naïve cells. In this respect, humoral and cellular immunity play a critical role in controlling both the primary [143, 144] and persistent [143, 145] phases of virus infection. In particular, EBV stimulates strong humoral responses to some lytic cycle proteins: IgM and developing IgG responses to nucleocapsid and envelope proteins are in fact detectable in primary EBV infection. Moreover, IgG responses to some of the immediate early and early lytic cycle proteins and to the latent protein EBNA1 and 2 are also usually detectable, together with neutralizing antibodies directed against gp350. Therefore, humoral responses play an important role in controlling the spread of the virus in the late phases of infection, while cellular immunity is fundamental in controlling both the primary and persistent phases of EBV propagation. More precisely, cytotoxic T lymphocytes (CTL) are the major determinants in the control of acute EBV infection [146, 147] and are directed against both lytic and latent antigens [148]. More than 44% of the total CD8^+^ T cells in acute infection are specific for a single lytic EBV epitope, and most of these epitope-specific cells have an activated/memory phenotype [149]. Conversely, in the late stages of infection, the frequency of epitope-specific CD8^+^ T cells selectively increases against latent EBV proteins, confirming that CTLs are the most important cells for limiting infection in the convalescent phase of virus infection [149].

## 4. Immunotherapy Approaches against EBV-Associated Malignancies

The development of malignancies associated with EBV is largely favored by an underlying defect in EBV-specific CTL immunity and function. Therefore, much work has been focused in the last years on the reconstitution of CTL immunity to EBV in transplanted patients, who are at increased risk to develop PTLD as a consequence of iatrogenic immune suppression. Moreover, recent data indicate that other EBV-associated diseases such as NPC, HL, BL, and chronic active EBV infections (CAEBV) can potentially be treated with immunotherapeutic approaches.

### 4.1. EBV-Targeting Vaccines in Virus-Associated Malignancies

To date, the experience with human EBV vaccines is limited [150, 151] and, although potentially useful to prevent EBV associated malignancies, vaccines providing life-long immunity against primary EBV infection might be unfeasible, considering that the type of immunity required to prevent repeated infection through mucosal surfaces is not clearly defined. Moreover, repeated infections with different EBV strains have been described [152], suggesting that the natural immune response to EBV is not sufficient to protect healthy EBV-positive individuals from recurrent infections. Nevertheless, it was clearly demonstrated that the risk of developing HL is 4-fold higher after resolution of IM, which is symptomatic of an abnormal primary EBV infection and then of a massive expansion of EBV-specific T cells [152]. Therefore, preventive vaccination to avoid uncontrolled virus replication and successive “scaring” of the immune system could decrease the incidence of EBV-associated malignancies. In particular, vaccine strategies for the immunotherapy of EBV-related tumors should seek to elicit or boost specific cellular immune response against EBV antigens expressed in these malignancies. Individuals likely to benefit from this approach are EBV-seronegative patients prior to solid organs transplant or patients with EBV-associated malignancies with a low tumor burden or in remission. However, vaccine strategies are unlikely to be the optimal method to enhance EBV-specific T-cell responses for patients who are immunocompromised, due to immunosuppressive therapies after transplantation or as a result of disease-related immune alterations such as in HL. In these cases, the adoptive immunotherapy with *ex vivo* activated EBV-specific CTL (EBV-CTLs) seems to be more promising, also because it allows genetic modifications of T cells able to enhance their function.

### 4.2. EBV-Specific Adoptive Immunotherapy (ACT) in PTLDs

EBV infection poses a significant problem in transplant patients who are iatrogenically immunosuppressed in order to prevent chronic organ rejection. Risk factors for the development of PTLD include EBV-seronegativity in the transplant recipient, the type of organ transplanted (highest in lung and heart and lowest in liver and kidney), and the level and type of immune suppression [27]. PTLD emerges as either of recipient or donor origin, depending on the type of transplant. For example, bone marrow transplant (BMT) patients develop PTLD of donor origin, as EBV-infected B cells derived from the donor marrow proliferate uncontrollably into lymphoma. Conversely, solid organ transplant patients develop PTLD of recipient origin, as EBV released from the transplanted organ infects the recipient's B cells [28]. On these grounds, initial studies investigated the potential of EBV-specific CTLs to treat PTLD in BMT patients, as CTLs could be easily generated from EBV-seropositive, immunocompetent donors. Pioneering studies [153, 154] demonstrated that PTLD was resolved after adoptive transfer of EBV-CTLs grown from donor peripheral blood mononuclear cells. The method developed to stimulate and expand large numbers of EBV-CTLs utilized donor's autologous EBV-immortalized lymphoblastoid B-cell lines (LCLs), which were cocultured with donor PBMCs in the presence of interleukin-2 (IL-2). Similar to PTLD tumor cells, LCLs also have a latency III phenotype and can activate polyclonal EBV-specific CTLs with a broad reactivity to a range of EBNA- and LMPs-derived epitopes. The resulting EBV-CTLs used in these studies killed donor LCLs *in vitro*, did not compromise allograft function, and most importantly, eradicated tumors [154].

### 4.3. EBV-Specific ACT in NPC and HL

Clinical evidence accumulated so far indicates that ACT with EBV-CTLs is safe and well tolerated. Even if this approach results particularly effective in the case of most immunogenic tumors, as previously described in PTLDs [155], in Latency II EBV-associated malignancies, the more restricted pattern of viral latent antigen expression, together with local immune suppression and immune evasion of tumor cells, strongly limit the therapeutic potential of EBV-CTLs obtained by conventional protocols. In particular, the infusion of EBV-targeted autologous CTLs was shown to enhance specific immune responses and to induce objective clinical responses only in a proportion of NPC and HL cases [156, 157], probably due to the weak immunogenicity of LMPs [67, 158]. On these grounds, recombinant viruses encoding for EBNA1, LMP1, and LMP2 antigens have been used to improve protocols for the *in vitro *expansion of EBV-CTLs and *in vivo* data showed that they were also able to protect against LMP-positive tumor growth in mice [159–161]. Accordingly, interesting results were recently obtained by Bollard and colleagues on 10 selected HL patients, by the induction and *in vivo* persistence of LMP2-specific CTLs, using genetically modified APCs [162]. The authors showed that 9 of 10 patients treated in remission of a high-risk disease remained in remission, and 5 of 6 patients with active relapsed disease had a tumor response, which was complete in 4 and sustained for more than 9 months [162]. Furthermore, these EBV antigen-specific effector lines are being tested, with promising results, also in NPC patients [158, 163–165]. Unfortunately, although LMP2-specific CTLs could be expanded *ex vivo* after peptide-pulsed DC injection in NPC patients [156], these responses were usually weak or transient. Learning from these trials and as a result of a better understanding of the crucial role for CD4^+^ T cells in assisting CD8^+^ T-cell immunity, more recent stimulation protocols aim to incorporate both CD4^+^ and CD8^+^ T cell antigens [65, 158]. Indeed, in addition to CD4^+^ T-cell help for CD8^+^ T-cell responses, CD4^+^ T cells can also target and kill EBV-transformed B cells directly [166]. Finally, considering that NPC and HL malignant cells have functional antigen-processing machinery and express HLA and costimulatory molecules [72, 167], the demonstration that other viral latent proteins expressed by these neoplastic cells may serve as tumor-associated antigens could provide the rational background to improve the clinical efficacy of ACT protocols in this setting. Particularly promising in this respect is the demonstration that the oncogenic EBV-encoded BARF1 protein, expressed by most NPCs, is immunogenic and thus may be exploited as additional target of innovative ACT protocols [168]. Therefore, ACT with EBV-CTLs has proven to be an effective strategy in the post-transplant setting, being able to reconstitute EBV-specific immunity [23], to prevent the development of PTLD [28] and treat patients with established PTLD. For other EBV-associated malignancies, the use of EBV-CTL has proven less efficacious; however, the results obtained so far are sufficiently encouraging to justify continued active exploration of this approach. Novel approaches are being developed to enhance the potency of EBV-specific immunotherapy by targeting CTL to subdominant EBV proteins and by genetically modifying these effector cells to render them resistant against inhibitory cytokines or immunosuppressive therapies [169, 170]. Notably, such strategies could have relevant implications for the ACT of a broader spectrum of human cancers with defined tumor antigens. All these approaches open promising avenues to enhance or prime protective EBV-specific immune responses, which have been suppressed by the tumor cells itself or by their microenvironment, and whose absence might predispose for the development of EBV-associated malignancies.

## 5. Innate Immunity-Based Therapies for EBV-Driven Tumors

Traditional approaches to exploit innate immunity for cancer immunotherapy include the activation of endogenous effectors (mainly NK cells) with systemic administration of cytokines (IL-2, etc.) and the adoptive infusion of *ex vivo* expanded NK or lymphokine-activated killer (LAK) cells. The potent immunostimulatory effect of systemic IL-2 in advanced cancer patients has been originally demonstrated by the seminal work of Rosenberg and collaborators [171, 172]. Since then, several clinical trials based on IL-2 administration have been carried out in different cohorts of cancer patients, mainly including melanoma, renal cell carcinoma, neuroblastoma, breast cancer, and several hematologic malignancies [173–177]. Notably, low-dose IL-2 was successfully used for the treatment or prevention of lymphomas associated with human immunodeficiency virus (HIV) infection, which include a significant proportion of EBV^+^ cases [178]. In these lymphoma patients, IL-2 treatment results in increased absolute numbers of NK cells, with no significant change in T-cell subsets or plasma HIV RNA levels [179]. Clinical experience however indicate that IL-2 can cause systemic toxicities, a limitation that could be overcome by the use of IL-15, a less toxic cytokine that plays essential roles in NK cell development, homeostasis, and survival [180]. Moreover, availability of clinical-grade IL-15 also opens new avenues to improve the generation of NK cells for adoptive cancer immunotherapy [181]. It should be considered, however, that the ability of NK cells to recognize and kill tumor cells may be attenuated with progression of the disease through several mechanisms, including the upregulation of negative regulators of immune responses, such as the ligand of Programmed Death-1 (PD-1) (PD-L1) [182, 183]. While the constitutive or inducible expression of PD-1 has been characterized in B, T, and dendritic cells, little is known regarding PD-1 expression on NK cells. Recent data indicate that the PD-1/PD-L1 signaling axis mediates NK cell activation and cytotoxicity against multiple myeloma. In particular, the use of a monoclonal antibody targeting the PD-1 molecule expressed by NK cells was shown to enhance NK cell cytotoxicity against tumor cells [184], providing the rationale to include these drugs in new therapeutic regimens for multiple myeloma. Several investigators have reported high levels of PD-1 expression on EBV-specific CD8^+^ T cells during acute or chronic infection [185, 186], but data on the expression of this inhibitory molecule on NK cells from patients with EBV-driven tumors are lacking.

Several lines of evidence indicate that innate immunity may usefully complement the antitumor immune responses induced by ACT. After total body irradiation, in fact, the damage of mucosal barriers results in the spreading of bacterial products, TLR agonists and proinflammatory cytokines that promote the activation of APC [187]. Moreover, it has been also demonstrated that TLR agonists may function as promising adjuvants for ACT protocols [188]. In a mouse melanoma model, TLR agonists were shown to enhance ACT in two functional phases: first, by favouring the interactions between activated host DCs and adoptively transferred CTLs; second, by enhancing the antitumor activity of T lymphocytes through IFN*γ*-dependent mechanisms [188]. This certainly constitutes a promising approach that deserves to be validated in humans. Besides working in combination with adoptively infused T cells, innate immunity effectors may be also used as a direct cell-based immunotherapy for human cancers, particularly those associated with oncogenic viruses. NK cells, in fact, can be an attractive alternative to ACT because they preferentially target MHC-class-I-deficient tumor cells. Moreover, NK cells can influence other immune cells by producing cytokines able to promote DC differentiation, antigen presentation, and CTL activity. Importantly, NK cells can eliminate immature, tolerizing DCs, allowing thus an improved presentation of tumor antigens to T cells. Pioneering studies by Velardi and coworkers demonstrated that alloreactive NK cells, if unrestrained by inhibitory signals from the recipient HLA ligands, are effective in protecting against disease relapse after hematopoietic stem cell transplantation [189]. Although available evidence indicates that alloreactive NK cells are effective in the setting of hematologic malignancies, this strategy remains to be extensively tested in patients with solid tumors, including EBV-associated carcinomas. Several methods are now available for *ex vivo* expansion of NK cells [190] and the critical issues related to the development of successful NK cell-based adoptive immunotherapy have been recently reviewed [191]. In the setting of EBV-driven tumors, exploitation of NK cells for therapeutic purposes seems particularly attractive, considering the pivotal role of these effectors in controlling EBV-infected cells. Moreover, IL-2-activated peripheral blood NK cells were shown to restrict EBV-induced B-cell transformation *in vitro* [86, 87] and activated NK cells acquire the ability to kill lytically EBV replicating B cells [192]. This latter effect was associated with downregulation of HLA class I molecules that bind to NK cell inhibitory receptors, whereas activating receptors remained upregulated [192]. In this respect EBV differs from human cytomegalovirus, which has evolved a complex and highly effective strategy for NK cell evasion. On these grounds, the current therapeutic approaches for EBV-related tumors based on the induction of EBV lytic replication would certainly benefit from a retained or even enhanced functionality of NK cells. It is worth considering in this respect that there is increasing interest in pharmacologic activation of lytic viral expression in EBV-driven malignancies [193]. Several drugs are able to trigger EBV lytic cycle, including gemcitabine, doxorubicin, various histone deacetylase inhibitors (trichostatin A, sodium butyrate, valproic acid, suberoylanilide hydroxamic acid), and bortezomib [193–195]. Particularly promising is the combination of EBV lytic cycle inducers with antiviral agents, as shown by the results of a phase I/II trial of arginine butyrate and gancyclovir in refractory EBV-associated lymphoid malignancies [196].

Cellular microRNAs (miRNAs) are emerging as new promising therapeutic targets for human cancers by virtue of their role in regulating numerous processes, also including immune responses [197]. In particular, recent advances have suggested new modalities to improve T-cell-based cancer immunotherapy by engineering distinct miRNAs. Targeting miR-17-92 is a promising approach considering that its downregulation in T cells from tumor-bearing mice and cancer patients is associated with a Th2 skewing and impaired antitumor responses [198]. Forced expression of miR-17-92 in T lymphocytes could therefore confer resistance to tumor-derived immunosuppressive factors, such as TGF-*β* and improve Th1 reactivity. Similarly, over-expression of miR-155 promotes Th1 differentiation of CD4^+^ T cells through the regulation of IFN-*γ*R*α* chain [199]. Moreover, it has been shown that miR-181 regulates the strength and sensitivity of TCR-mediated T-cell activation by acting through modulation of multiple phosphatases [200]. It would be relevant to assess whether ectopic expression of miR-181 would lead to enhanced TCR signaling and a reduced T-cell activation threshold in T cells recognizing weak tumor-associated antigens. While the expression and importance of miRNAs in T and B lymphocytes have been established, little is known about the role of miRNAs in NK cells. The recent characterization of NK cell miRNA transcriptome by next-generation sequencing will provide a valuable framework to identify critical miRNAs that can be targeted to improve NK cell function in cancer patients [201]. Considering that NK cells also share an miRNA profile with effector and memory CD8^+^ T cells [202], the possibility to target miRNAs regulating pathways common to innate and adaptive immunity appears particularly attractive. miRNAs expressed by EBV-infected tumor cells may be also relevant for therapeutic purposes. In fact, cellular miRNAs 200b and 429 are key regulators of the switch between latent and lytic EBV infection, thus constituting attractive targets for the development of new lytic induction therapeutics [203].

## 6. Concluding Remarks

Innate and adaptive immunity are both crucial for an effective control of an oncogenic virus such as EBV. The success of adoptive T-cell therapy for EBV-driven PTLDs is stimulating the development of new immunotherapeutic strategies for EBV-associated tumors. Considering that adaptive immunity has coevolved with innate immunity to warrant protection from infections, innovative immunotherapeutic approaches should take into account both compartments to be more clinically effective. Evidence accumulated so far indicates that innate immunity effectors, particularly NK cells, can be exploited with therapeutic purposes and new targets have been recently identified. Nevertheless, only few clinical studies are investigating the possible efficacy of treatments exploiting both innate and adaptive antitumor immunity. EBV-driven tumors constitute a particularly suitable setting to address this clinically relevant issue.

##  Authors' Contribution

D. Martorelli and E. Muraro equally contributed to the work.
